# Accumulation of Glucosylceramide in the Absence of the Beta-Glucosidase GBA2 Alters Cytoskeletal Dynamics

**DOI:** 10.1371/journal.pgen.1005063

**Published:** 2015-03-24

**Authors:** Diana Raju, Sophie Schonauer, Hussein Hamzeh, Kevin C. Flynn, Frank Bradke, Katharina vom Dorp, Peter Dörmann, Yildiz Yildiz, Christian Trötschel, Ansgar Poetsch, Bernadette Breiden, Konrad Sandhoff, Heinz G. Körschen, Dagmar Wachten

**Affiliations:** 1 Minerva Research Group—Molecular Physiology, Center of Advanced European Studies and Research, Bonn, Germany; 2 Axon Growth and Regeneration, German Center for Neurodegenerative Diseases (DZNE e.V.), Bonn, Germany; 3 Institute of Molecular Physiology and Biotechnology of Plants, University of Bonn, Bonn, Germany; 4 Innere Medizin am Landeskrankenhaus Bregenz, Bregenz, Austria; 5 Biochemie der Pflanzen, Ruhr-Universität Bochum, Bochum, Germany; 6 Life and Medical Sciences Institute (LIMES) c/o Kekulé-Institute of Chemistry and Biochemistry, Bonn, Germany; 7 Department of Molecular Sensory Systems, Center of Advanced European Studies and Research, Bonn, Germany; Stanford University School of Medicine, UNITED STATES

## Abstract

Glycosphingolipids are key elements of cellular membranes, thereby, controlling a variety of cellular functions. Accumulation of the simple glycosphingolipid glucosylceramide results in life-threatening lipid storage-diseases or in male infertility. How glucosylceramide regulates cellular processes is ill defined. Here, we reveal that glucosylceramide accumulation in GBA2 knockout-mice alters cytoskeletal dynamics due to a more ordered lipid organization in the plasma membrane. In dermal fibroblasts, accumulation of glucosylceramide augments actin polymerization and promotes microtubules persistence, resulting in a higher number of filopodia and lamellipodia and longer microtubules. Similar cytoskeletal defects were observed in male germ and Sertoli cells from GBA2 knockout-mice. In particular, the organization of F-actin structures in the ectoplasmic specialization and microtubules in the sperm manchette is affected. Thus, glucosylceramide regulates cytoskeletal dynamics, providing mechanistic insights into how glucosylceramide controls signaling pathways not only during sperm development, but also in other cell types.

## Author Summary

During mammalian spermatogenesis, sperm with a head and a tail are formed from a round cell. This process is tightly regulated and involves the close interaction of somatic Sertoli cells and germ cells. Accumulation of the glycosphingolipid glucosylceramide in the absence of the beta-glucosidase GBA2 has been proposed to disturb sperm development, leading to morphological defects. However, the underlying mechanism is not known. Here, we demonstrate that accumulation of glucosylceramide in GBA2 knockout-mice controls the dynamics of the actin and microtubule cytoskeleton, which are crucial for sperm development. In particular, cytoskeletal structures at the interface between Sertoli and germ cells are disorganized, leading to malformation of the sperm head and a defect in acrosome formation. In summary, we provide mechanistic insights into how glucosylceramide controls cellular signaling and dysregulation of this essential glycosphingolipid leads to male infertility.

## Introduction

Spermatogenesis occurs in the seminiferous tubules of the testis. Defects in sperm development often result in male infertility. The beta-glucosidase GBA2 plays an important role in sperm development [1]. GBA2 knockout-mice are subfertile, because sperm display severe morphological defects: heads are round rather than sickle-shaped, mitochondria are misaligned along the sperm flagellum, and the acrosome, needed to penetrate the egg coat, is lacking [1]. This phenotype is called globozoospermia [2]. GBA2 degrades the glycosphingolipid glucosylceramide (GlcCer) to glucose and ceramide. Accumulation of GlcCer in GBA2 knockout-mice has been proposed to underlie the defects in spermatogenesis leading to globozoospermia [1]. However, the underlying mechanism is not known.

Several knockout-mouse models display globozoospermia. In some models, vesicle fusion leading to acrosome formation is impaired [3–10]. The acrosome is a large, Golgi-derived vesicle that is tethered to the nuclear envelope [11]. The acrosome is formed in round and elongated spermatids [12,13] through budding of vesicles from the trans-Golgi network (TGN). These vesicles are transported to the nuclear envelope, where they fuse to form a single acrosomal vesicle [12,13]. However, other globozoospermia-related proteins are not involved in vesicular transport, but rather in acrosomal anchoring to the nuclear envelope or condensation of the sperm nucleus [14–16].

During spermiogenesis, spermatids undergo dramatic morphological changes, which occur while the cells are transported across the seminiferous epithelium into the lumen. The transport depends on the close interaction between developing sperm and Sertoli cells [17,18]. Actin bundles emanating from Sertoli cells into the ectoplasmic specialization (ES), a testis-specific adherens junction, undergo extensive re-organization while they break-down and reassemble to transport the developing sperm to the lumen [19,20]. A podosome-like structure, the so-called tubulobulbar complex, forms between spermatids and Sertoli cells; it internalizes intact junctions during sperm development and positions the developing sperm cell during the transit through the seminiferous tubules [21,22]. A bundle of filamentous actin (F-actin), which emanates from Sertoli cells, embraces each tubulobulbar complex; this interaction connects the endoplasmic reticulum (ER) of Sertoli cells to the tubulobulbar complex of spermatids [17]. Furthermore, the spermatid manchette, a microtubule-based structure that is transiently formed also contributes to shaping of the sperm head [23]. The manchette consists of a perinuclear microtubule ring. During spermatid elongation, this ring constricts to decrease the diameter of the elongating spermatid head [23].

Here, we demonstrate that cytoskeletal dynamics controlling sperm-head shaping and acrosome formation are affected by accumulation of GlcCer in GBA2 knockout-mice, which results in globozoospermia and, thereby, male infertility.

## Results

### GBA2 is expressed in Sertoli cells

**Fig 1:**
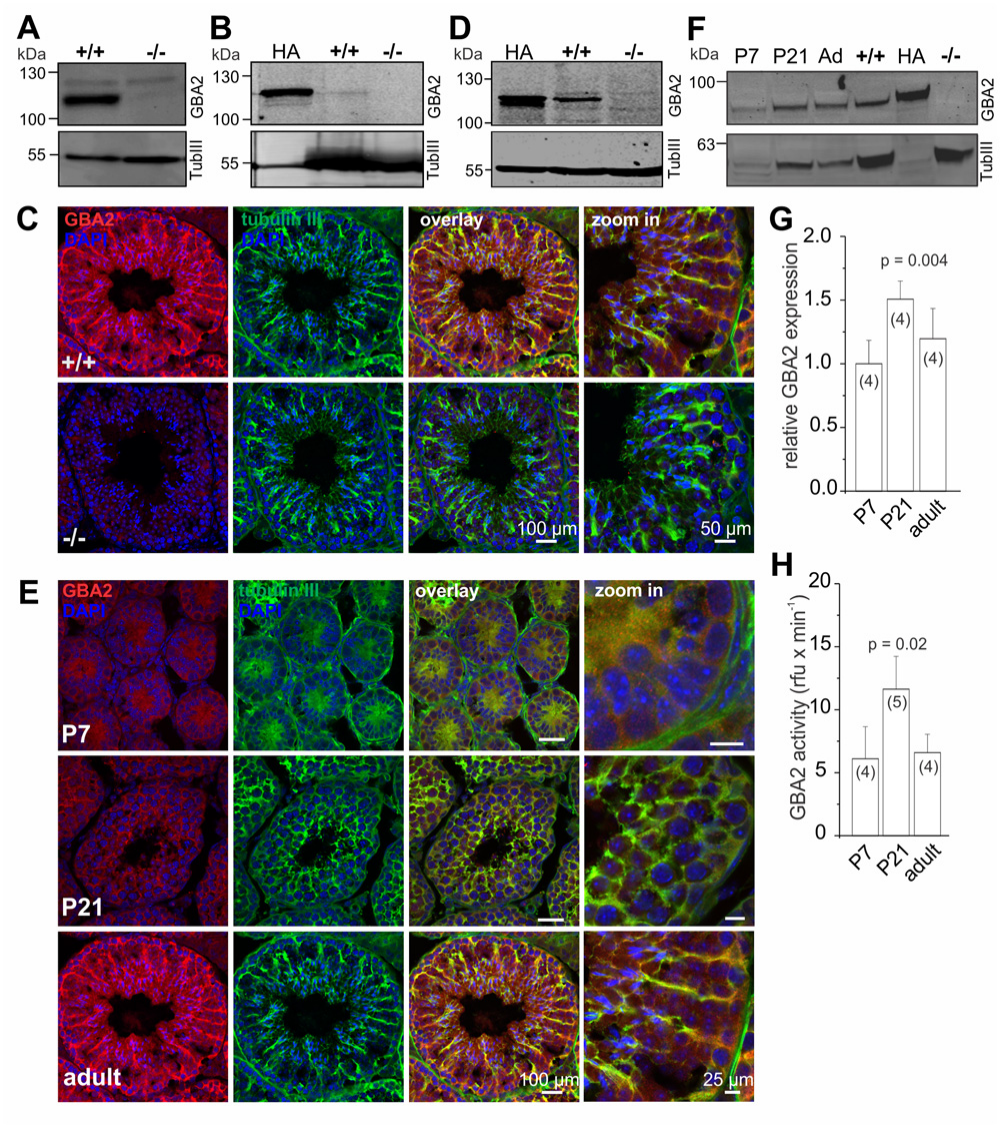
GBA2 is expressed in Sertoli cells. **(A)** GBA2 expression in testis. Total protein lysates were probed with a GBA2-specific antibody (2F8) on a Western blot. Beta-tubulin was used as a loading control. +/+: wild-type;-/-: GBA2 knockout. **(B)** GBA2 expression in mouse sperm. Total protein lysates were probed with a GBA2-specific antibody (2F8) on a Western blot. Heterologously expressed HA-tagged GBA2 was used as a positive control, beta-tubulin as a loading control. **(C)** Immunohistochemical analysis of GBA2 in testis. Testis cross-sections were labeled with a GBA2-specific antibody (pcGBA2, red) and an anti-beta tubulin III antibody (green) as a marker for Sertoli cells. DAPI was used to label the DNA. Scale bars are indicated. **(D)** GBA2 expression in P7 Sertoli cells. See (B). **(E-G)** GBA2 expression during development. **(E)** See (C) for testis cross-sections from P7, P21, and adult wild-type mice. **(F)** Representative Western blot using protein lysates from wild-type testis at P7, P21, and adult mice (ad). Heterologously expressed HA-tagged GBA2 has been used as a positive control and beta-tubulin III as a Sertoli-cell marker (TubIII). Protein lysates from wild-type (+/+) and GBA2 knockout-brain (-/-) are shown as controls. **(G)** Quantification of GBA2 protein expression. Expression levels of GBA2 have been normalized to beta-tubulin III. Data are presented as mean ± S.D.; n numbers and p values calculated using One-Way ANOVA are indicated. **(H)** GBA2 activity during sperm development. Beta-glucosidase activity was measured in protein lysates from wild-type testis at pH 6 using 1.67 mM of the artificial substrate 4-methylumbelliferyl-beta-D-glucopyranoside. Data are presented as mean ± S.D.; n numbers and p values calculated using One-Way ANOVA are indicated.

To investigate the role of GBA2 during spermatogenesis, we analyzed GBA2 expression in the testis (Fig. 1A). Although the main defect in GBA2 knockout-mice occurs in sperm, GBA2 was only weakly if at all expressed in sperm (Fig. 1B). In fact, also mass spectrometry failed to detect peptides derived from GBA2 in mouse sperm. However, three lines of evidence demonstrate that GBA2 is expressed in Sertoli cells. First, immunofluorescent labeling of testis sections demonstrated co-localization of GBA2 and beta-tubulin III, a Sertoli cell marker (Fig. 1C). Second, Western blot-analysis of Sertoli cells, isolated from P7 old males, when the seminiferous epithelia consist exclusively of Sertoli cells and type A spermatogonia [24], showed that GBA2 is expressed in Sertoli cells (Fig. 1D). Finally, mass spectrometry analysis identified four and two unique GBA2 peptides in testis and Sertoli cells, respectively (S1 Fig). In summary, GBA2 is predominantly expressed in Sertoli cells.

In the mammalian testis, sperm are continuously produced throughout the reproductive period. Although spermatogenesis is precisely controlled, only the first spermatogenic wave is synchronized and, therefore, allows assigning the expression of a protein to the respective developmental stage. Thus, we analyzed the time course of GBA2 expression and activity during the first spermatogenic wave (P7: pre-puberty, P21: early puberty) and in adult testis (> 22 weeks). GBA2 followed the expression pattern of beta-tubulin III during the first spermatogenic wave (Fig. 1E). When normalizing the expression of GBA2 to beta-tubulin III, GBA2 expression increased from P7 to P21 and decreased again from P21 to adult (Fig. 1F, G). In parallel, GBA2 activity increased from P7 to P21 and decreased from P21 to adult (Fig. 1H), suggesting that GBA2 plays an important role during the first wave of spermatogenesis.

### GlcCer accumulates in testis and sperm of GBA2 knockout mice

**Fig 2:**
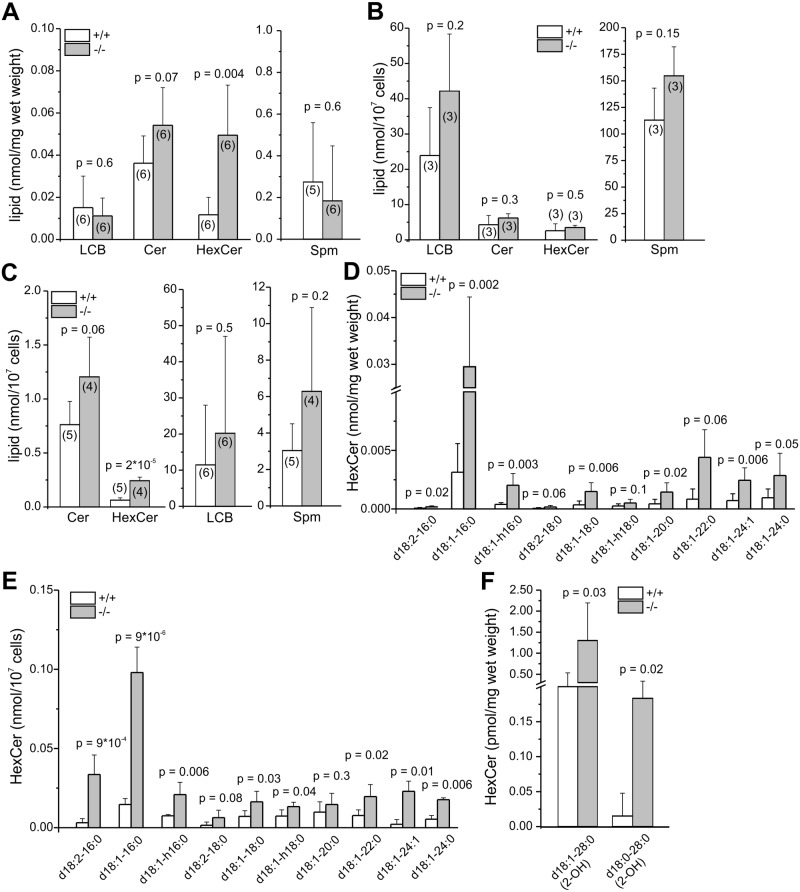
Lack of GBA2 results in accumulation of GlcCer. **(A)** Quantitative analysis of neutral sphingolipids in adult testis. +/+: wild-type;-/-: GBA2 knockout. LCB: long-chain bases, Cer: ceramides, HexCer: hexosylceramides, Spm: sphingomyelins. **(B)** See (A) for P7 Sertoli cells. **(C)** See (A) for sperm. **(D)** Quantitative analysis of HexCer in adult testis. Lipids are classified according to their acyl chain-length. **(E)** See (D) for sperm. **(F)** See (D) for very long chain fatty acids. All data are presented as mean ± S.D.; n numbers and p values calculated using One-Way ANOVA are indicated.

The fertility defect of GBA2 knockout-mice has been attributed to the accumulation of GlcCer in testis [1]. Germ and Sertoli cells contain a plethora of different glycosphingolipids (GSLs) [25]. Whereas germ cells contain glycosphingolipids (fucosylated GSLs, GSLs), ceramides (Cer), and sphingomyelings (Spm) with polyenoic very long-chain fatty acids, Sertoli cells predominantly harbor sphingolipids with saturated long-chain fatty acids [25]. We analyzed the amount of neutral sphingolipids in testis, Sertoli cells (P7), and sperm from wild-type and GBA2 knockout-mice by mass spectrometry and distinguished between sphingoid bases (long-chain bases, LCB; C_18_), ceramides (different chain length, saturated and unsaturated), hexosylceramides (HexCer; different chain length, saturated and unsaturated), and sphingomyelin (different chain length, saturated and unsaturated). In testis from GBA2 knockout-mice, total HexCer levels were dramatically increased, whereas total levels of LCB, Cer, and Spm were not affected (Fig. 2A). Using TLC (thin-layer chromatography), it has already been shown that in brain, only glucosylceramide, but not galactosylceramide accumulates in GBA2 knockout-mice [1]. Thus, the increase in HexCer levels can be attributed to an increase in GlcCer levels. Sertoli cells at P7 did not show a change in any of the sphingolipids (Fig. 2B), whereas in sperm, similar to testis, the GlcCer levels were increased (Fig. 2C). In both testis and sperm, C16 GlcCer levels were most dramatically increased, but also C18, C20, C22, and C24 GlcCer levels were elevated (Fig. 2D, E). In sperm, levels of long-chain GlcCer were similar between wild-type and GBA2 knockout-mice, whereas in testis, the C28 GlcCer levels were increased, indicating an accumulation in Sertoli cells (Fig. 2F). Thus, GlcCer accumulates in testis and sperm from adult GBA2 knockout-mice (˃ 22 weeks), but not in Sertoli cells at P7.

### Loss of GBA2 results in cytoskeletal defects in the testis

**Fig 3:**
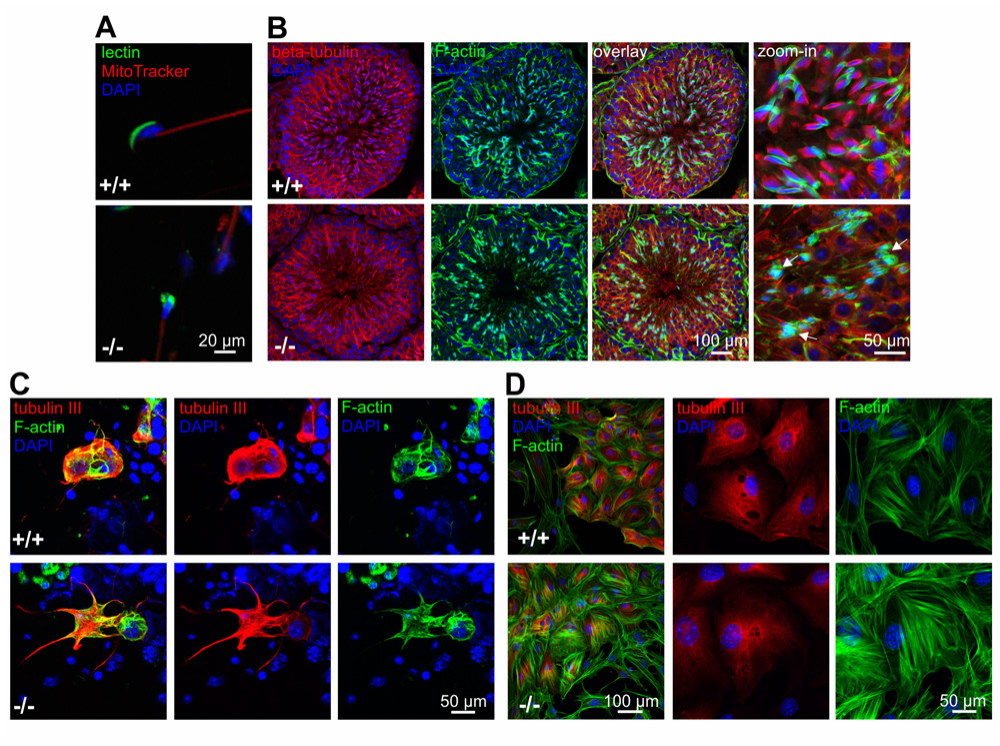
Lack of GBA2 causes globozoospermia and cytoskeletal defects in the testis. **(A)** Immunofluorescent labeling of wild-type (+/+) and GBA2 knockout-sperm (-/-). Fluorescent peanut lectin (green), a mitotracker (red), and DAPI (blue) was used to label the acrosome, the mitochondria in the sperm flagellum, and the DNA, respectively. Scale bars are indicated. **(B)** Immunofluorescent labeling of the cytoskeleton in adult wild-type (+/+) and GBA2 knockout-testis (-/-). Microtubules were labeled using an anti-beta tubulin antibody (red), F-actin using Alexa Fluor 488 Phalloidin (green), and the DNA using DAPI (blue). Defects in the F-actin structure are highlighted with arrows. Scale bars are indicated. **(C)** Immunofluorescent labeling of germ and Sertoli cells isolated from adult wild-type (+/+) and GBA2 knockout-testis (-/-). Cells were labeled with an anti-beta tubulin III antibody (red) as a marker for Sertoli cells, Alexa Fluor 488 Phalloidin (green) to label F-actin, and DAPI to label the DNA (blue). Scale bars are indicated. **(D)** See (C) for P7 Sertoli cells.

Sperm from GBA2 knockout-mice display globozoospermia [1]: the heads are round rather than sickle-shaped and the acrosome is malformed (Fig. 3A). However, the underlying molecular mechanisms are not known. The cytoskeleton plays a major role during spermatogenesis, in particular in regulating the interaction between Sertoli and germ cells at the ES and in shaping the sperm head [20,23,26,27]. Using fluorescence microscopy, we investigated the distribution of the actin and microtubule cytoskeleton in the testis of adult wild-type and GBA2 knockout-mice (Fig. 3B): in wild-type testis, F-actin hoops in the ES displayed a sickle-shape alignment around sperm heads, whereas in GBA2 knockout-mice, F-actin accumulated in the ES and was misaligned around the round sperm heads (Fig. 3B). However, the microtubule network appeared rather normal (Fig. 3B).

To analyze the cytoskeletal structures on a single cell level, we isolated Sertoli and germ cells from adult wild-type and GBA2 knockout-testis. In isolated cells, both the microtubule and F-actin network appeared more extensive in Sertoli cells from GBA2 knockout-mice with increased microtubule bundling and protrusive F-actin structures (Fig. 3C).

Together, our results show that cytoskeletal defects occur in particular in Sertoli cells, where GBA2 is predominantly expressed.

**Fig 4:**
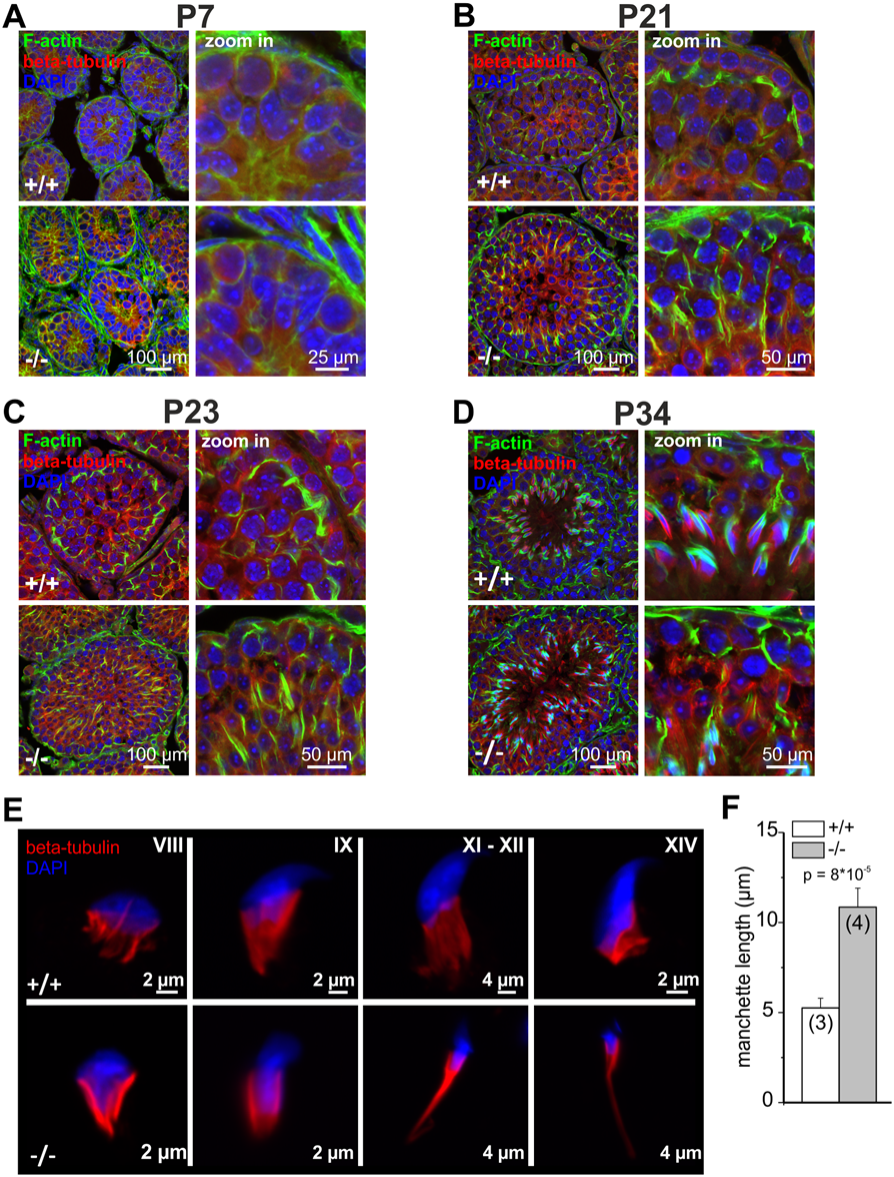
Cytoskeletal defects in GBA2 knockout-testis already occur during the first spermatogenic wave. **(A)** Immunofluorescent labeling of the cytoskeleton in wild-type (+/+) and GBA2 knockout-testis (-/-) at P7. Microtubules have been labeled using an anti-beta tubulin III antibody (red), F-actin using Alexa Fluor Phalloidin 488 (green), and the DNA using DAPI (blue). Scale bars are indicated. **(B)** See (A) for P21. **(C)** See (A) for P23. **(D)** See (A) for P34. **(E)** Development of the manchette in spermatids. The manchette was stained with beta-tubulin (red), DNA was labeled with DAPI (blue). Different developmental stages are indicated. **(F)** Manchette length. The manchette length of spermatids from wild-type (+/+) and GBA2 knockout-mice (-/-) was determined using ImageJ. At least 30 cells have been analyzed per genotype. Data are shown as mean ± S.D.; n numbers and p values calculated using One-Way ANOVA are indicated.

In testis, GBA2 expression and activity increased from P7 to P21. To correlate the expression and activity of GBA2 in wild-type mice with the cytoskeletal defects in GBA2 knockout-mice, we determined the developmental time course of the cytoskeletal defects. At P7, Sertoli cells from GBA2 knockout-mice did not show any obvious defect in the F-actin or microtubule structures (Fig. 3D), underlining the results from the lipidomics and indicating that P7 might be a too early time point during spermatogenesis for defects to occur. To follow the first spermatogenic wave in more detail, we analyzed the organization of the cytoskeleton in the testis at different stages until P34, when the first wave terminates. Until P21, no difference in the microtubule or actin cytoskeleton was observed between wild-type and GBA2 knockout-mice (Fig. 4A, B). However, at P23, the alignment of F-actin in GBA2 knockout-testis started to change and at P34, the F-actin structures around the sperm heads were misaligned and sperm organization inside the testis lumen was disturbed (Fig. 4C, D). Thus, the occurrence of cytoskeletal defects in GBA2 knockout-mice correlates with the time course of GBA2 expression and activity in wild-type mice.

### Formation of the microtubule manchette is altered in GBA2 knockout-spermatids

Although GBA2 is predominantly expressed in Sertoli cells, GlcCer also accumulates in sperm from GBA2 knockout-mice (Fig. 2C). Thus, we hypothesized that the sperm cytoskeleton is also affected by accumulation of GlcCer. Abnormal sperm-head morphology has been linked to defects in the microtubule manchette [28–31]. The manchette is a microtubule structure that appears during the elongation process of spermatids [29,32,33], which first surrounds the proximal tip of the nucleus, gradually migrates towards the caudal end, and creates the force that is needed to shape the sperm head [34]. To analyze the formation of the microtubule manchette in wild-type and GBA2 knockout-spermatids, we monitored its formation in single, isolated germ cells (Fig. 4E). The microtubule manchette of wild-type spermatids was symmetric and conical-shaped, whereas the manchette of knockout spermatids was much more elongated. The manchette length of GBA2 knockout-spermatids was about twofold longer compared to wild-type spermatids (Fig. 4F;-/-: 10.9 ± 1.0 μm and +/+: 5.3 ± 0.5 μm, respectively). Thus, accumulation of GlcCer in the absence of GBA2 affects both the microtubule cytoskeleton of Sertoli cells and developing sperm.

### Temporal control of acrosome formation in GBA2 knockout-sperm is disturbed

**Fig 5:**
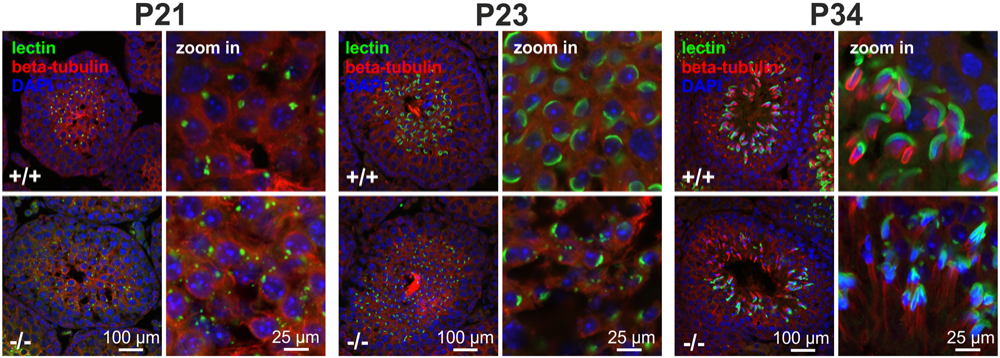
Acrosome development. Immunofluorescent labeling of the acrosome using fluorescent peanut lectin (green) in P21, P23, and P34 testes. Microtubules were labeled with an anti-beta tubulin antibody (red), DAPI (blue) has been used to stain the DNA. Scale bars are indicated.

During sperm development, the cytoskeleton plays a crucial role in forming the acrosome [35]. Acrosome formation and the appearance of the sperm manchette start at the same time [34]. Immunofluorescent analysis of GBA2 knockout-sperm revealed that the formation of the acrosome is disrupted (Fig. 3A) [1]. We therefore analyzed the acrosome formation during the first spermatogenic wave (Fig. 5). The acrosome is derived from vesicles emanating from the TGN [12,13] and its formation can be divided into different phases: Golgi phase, cap phase, and acrosomal phase. In the Golgi phase, vesicles from the TGN fuse at one pole of the nucleus to form a single large vesicle that is attached to the nucleus. In the cap phase, the vesicle flattens over the nuclear membrane, forming a cap-like structure around the spermatid head. In the acrosome phase, the cap-like structures stretches out to form the final acrosome [36]. During the Golgi phase (P21), lectin staining revealed that vesicles in wild-type spermatids were completely fused and polarized, whereas in GBA2 knockout-spermatids, vesicles were still dispursed throughout the cell and not fused at one end of the cell (Fig. 5). This defect was even more prominent in the cap (P23) and the acrosome phase (P34, Fig. 5). The acrosomal cap at P23 was incomplete and at P34, only a few GBA2 knockout-spermatids showed an acrosomal-like structure. Furthermore, the nuclear morphology was altered, leaving sperm with round heads at P34 (Fig. 5). Thus, the cytoskeletal defects in developing sperm and Sertoli cells and the defect in acrosome formation occur in parallel in GBA2 knockout-mice.

### Accumulation of GlcCer augments actin polymerization

**Fig 6:**
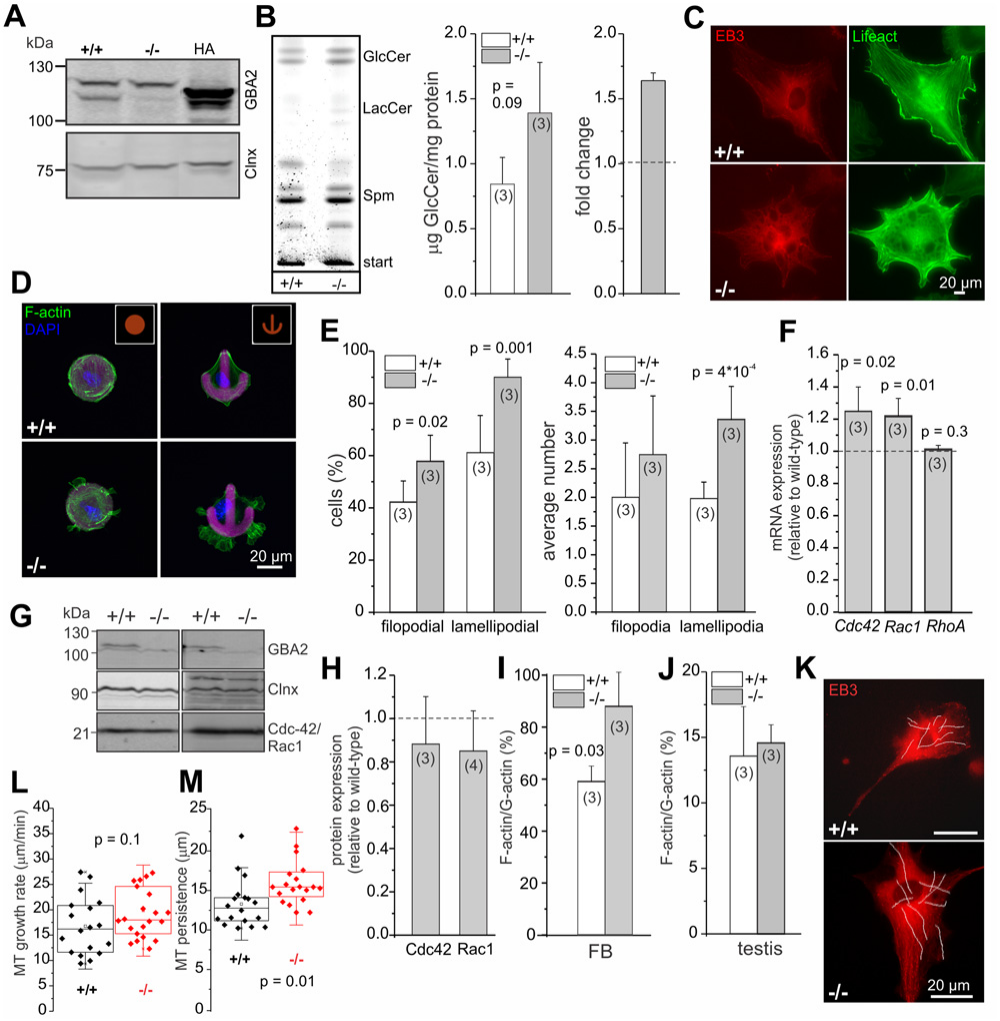
Dermal fibroblasts from GBA2 knockout-mice also display cytoskeletal defects. **(A)** GBA2 expression in dermal fibroblasts from adult mice. Total protein lysates were probed with a GBA2-specific antibody (2F8) on a Western blot. Heterologously expressed HA-tagged GBA2 was used as a positive control, calnexin (Clnx) as a loading control. **(B)** Accumulation of GlcCer in GBA2 knockout-fibroblasts. Thin layer chromatography (TLC) analyzing glycosphingolipids from wild-type (+/+) and GBA2 knockout-fibroblasts (-/-). Representative TLC analysis for neutral sphingolipids. GlcCer: glucosylceramide, LacCer: lactosylceramide, Spm: sphingomyelin. GlcCer levels were quantified by densitometry and are presented as mean ± S.D. The fold change in GlcCer levels in GBA2 knockout-fibroblasts was calculated. **(C)** Fluorescent labeling of the cytoskeleton in dermal fibroblasts from wild-type (+/+) and GBA2 knockout-mice (-/-). Cells were transfected with lifeact (green) to visualize F-actin and with EB3-cherry to visualize microtubules. Scale bars are indicated. **(D)** Fluorescent labeling of F-actin in dermal fibroblasts from wild-type (+/+) and GBA2 knockout-mice (-/-). Cells were seeded on CYTOO chips with micropatterns that are coated with fluorescently-labeled fibronectin (purple). F-actin was stained using Alexa Fluor Phalloidin 488 (green) and the DNA was stained with DAPI (blue). Scale bars are indicated. **(E)** Analysis of cytoskeletal structures. Cells were seeded on the crossbow shape. The number of cells containing filopodia or lamellipodia (left) and the average number of filopodia or lamellipodia per cell (right) were determined. **(F)** Gene expression-analysis. The mRNA expression level of *Cdc42*, *Rac1*, and *Rho* was analyzed by qRT-PCR. **(G)** Protein expression-analysis. Total protein lysates were probed with a GBA2- (2F8), a Cdc42-, and a Rac1-specific antibody on a Western blot. Calnexin (Clnx) was used as a loading control. **(H)** Quantification of protein expression based on (G). **(I)** Quantification of actin turnover in dermal fibroblasts. Expression levels of G- and F-actin in wild-type (+/+) and GBA2 knockout-fibroblasts (-/-) were determined using Western blot-analysis. Ratio of F-actin/G-actin for wild-type and GBA2 knockout-fibroblasts is expressed relative to the control. **(J)** See (I) for testis. **(K-M)** Analysis of microtubule dynamics in dermal fibroblasts from wild-type (+/+) and GBA2 knockout-mice (-/-). **(K)** Expression of EB3-cherry in dermal fibroblasts. Cells were transfected with EB3-cherry and microtubule dynamics were analyzed. Representative tracks of growing microtubule plus-ends are indicated with white lines. **(L)** Microtubule growth rate. Wild-type (+/+) and GBA2 knockout-fibroblasts (-/-) were transfected with EB3-cherry and the growth rate of growing plus-ends was analyzed. Per genotype, n = 3 animals with a minimum of 7 cells and 10 tracks per cell were analyzed. Data are presented as mean ± S.D. **(M)** see (L) for microtubule persistence. For all bar graphs, data are shown as mean ± S.D.; n numbers and p values calculated using One-Way ANOVA are indicated.

Germ and Sertoli cells from adult testis are difficult to culture and to manipulate. In contrast, primary fibroblasts are easy to isolate and to maintain in culture. To gain a more mechanistic insight into how accumulation of GlcCer affects cytoskeletal dynamics, we analyzed dermal fibroblasts from adult wild-type and GBA2 knockout-mice as a model system. Wild-type fibroblasts express GBA2 (Fig. 6A) and GBA2 knockout-fibroblasts accumulate GlCer in the absence of GBA2 (Fig. 6B). Indeed, the F-actin and microtubule network appeared strikingly different in GBA2 knockout compared to wild-type fibroblasts, which resulted in a dramatic change in cell morphology—knockout cells were more filopodia-like with extensive protrusions emanating from the plasma membrane (Fig. 6C). However, when maintained in culture, dermal fibroblasts are morphologically heterogeneous, which makes it difficult to analyze cytoskeletal structures in detail. Thus, we seeded fibroblasts on chips that were coated with specific patterns (crossbow, disc, Y-shape, dumb-bell) to force the cells into a given shape. Wild-type fibroblasts fully adhered to the given fibronectin pattern, whereas GBA2 knockout-fibroblasts did not completely align with a given pattern and showed extensive F-actin structures protruding from the cell membrane (Fig. 6D). This was particularly evident for the crossbow and disc shape (Fig. 6D). Cells mainly contain three different F-actin structures: filopodia, lamellipodia, and stress fibers. We compared the different actin structures between wild-type and GBA2 knockout-fibroblasts on the crossbow shape and observed that GBA2 knockout-fibroblasts were more prone to develop filopodia and lamellipodia compared to wild-type cells, while stress fiber formation appeared normal (Fig. 6D, E). Furthermore, the average number of lamellipodia per cell was significantly increased in GBA2 knockout compared to wild-type fibroblasts (3.35 ± 0.58 vs 1.98 ± 0.29, respectively; Fig. 6E).

Actin dynamics are prominently regulated by three members of the Rho family of small GTPases [37]. The formation of filopodia is controlled by Cdc42, whereas lamellipodia formation depends on the activity of Rac1 and stress fiber formation on the activity of RhoA [38–42]. First, we analyzed the expression of Cdc42, Rac1, and RhoA on the mRNA level using qRT-PCR. The expression of RhoA was similar in wild-type and GBA2 knockout-fibroblasts, whereas the expression of both Rac1 and Cdc42 was slightly increased (1.22 ± 0.11 and 1.24 ± 0.15, respectively; Fig. 6F). However, this was not reflected on the protein level: neither the expression level of Rac1 nor of Cdc42 was different between wild-type and GBA2 knockout-fibroblasts (Fig. 6G, H). Thus, a change in the expression level of these key proteins controlling actin dynamics does not underlie the cytoskeletal defects observed in GBA2 knockout-fibroblasts.

Lamellipodia and filopodia formation is induced by actin polymerization [41]. To study the impact of GlcCer accumulation on actin polymerization, we determined the G- and F-actin content and calculated the F-actin/G-actin ratio as a read-out for actin polymerization. The F-actin/G-actin ratio was significantly higher in GBA2 knockout-fibroblasts compared to wild-type fibroblasts, indicating that actin polymerization was augmented in GBA2 knockout-fibroblasts (Fig. 6I). Although actin turnover in testis from adult GBA2 knockout-mice was similar to wild-type mice, we could clearly show that F-actin structures in the ES are augmented (Fig. 6J, 3B). This is probably due to the heterogeneity of cells in adult testis, which makes it difficult to detect a change in F-/G-actin in a particular subcellular structure like the ES.

Taken together, our results reveal that accumulation of GlcCer induces actin polymerization without changing the expression of RhoA, Rac1, or Cdc42.

### Accumulation of GlcCer in dermal fibroblasts facilitates microtubule polymerization

An increase in the length of the manchette in GBA2 knockout-spermatids indicates that microtubules persist longer. Thus, we analyzed microtubule dynamics in wild-type and GBA2 knockout-fibroblasts. Cells were transfected with EB3-cherry, a fluorescent probe that binds to the plus-ends of growing microtubules and allows following microtubule assembly using live-cell imaging (Fig. 6K) [43]. Growing plus-ends appear as shooting comets, which allows determining the microtubule growth rate and persistence (Fig. 6L, M). Microtubule growth rate was not different between genotypes, but microtubules in GBA2 knockout-fibroblasts persisted significantly longer compared to wild-type fibroblasts (Fig. 6L, M). This is in line with the finding that microtubules in the manchette from GBA2 knockout-spermatids appear longer (Fig. 4E, F).

### Accumulation of GlcCer accelerates cell migration

**Fig 7:**
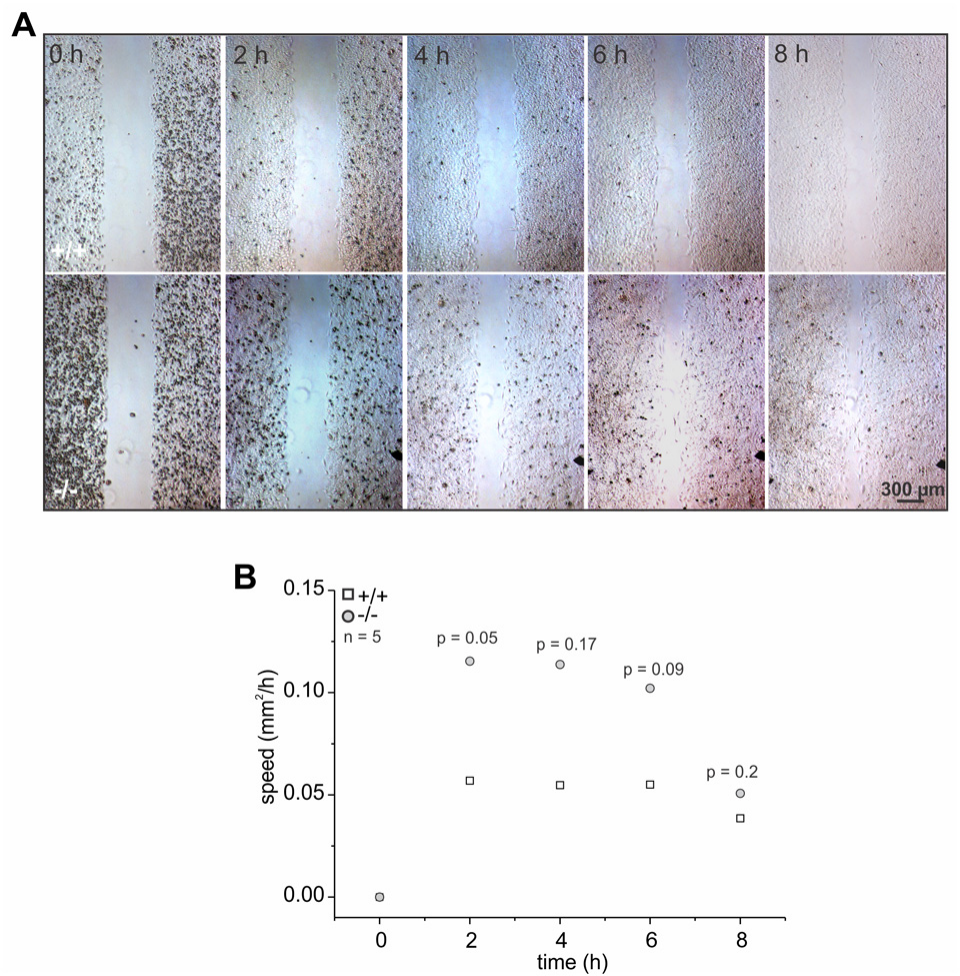
The absence of GBA2 affects cell migration. **(A-B)** Wound-healing assay to analyze cell migration of dermal fibroblasts from wild-type (+/+) and GBA2 knockout-mice (-/-). **(A)** Representative images at different time points after initiating the assay. Scale bars are indicated. **(B)** Analysis of cell migration. The rate of cell migration has been analyzed. Average data points for wild-type (+/+) and GBA2 knockout-fibroblasts (-/-) for different time points are shown; n numbers and p values using One-Way ANOVA are indicated.

In fibroblasts, microtubule polymerization can induce actin polymerization and, together, promote cell migration [44]. Thus, we analyzed the migration of wild-type and GBA2 knockout-fibroblasts in a wound-healing assay. GBA2 knockout-fibroblasts migrated faster than wild-type cells, in particular 2 to 6 h after starting the assay (Fig. 7A, B). Thus, loss of GBA2 followed by an accumulation of GlcCer promotes microtubule and actin polymerization, which in turn changes cellular behavior.

### Inhibition of GBA2 with NB-DNJ also affects cytoskeletal dynamics and cell migration

**Fig 8:**
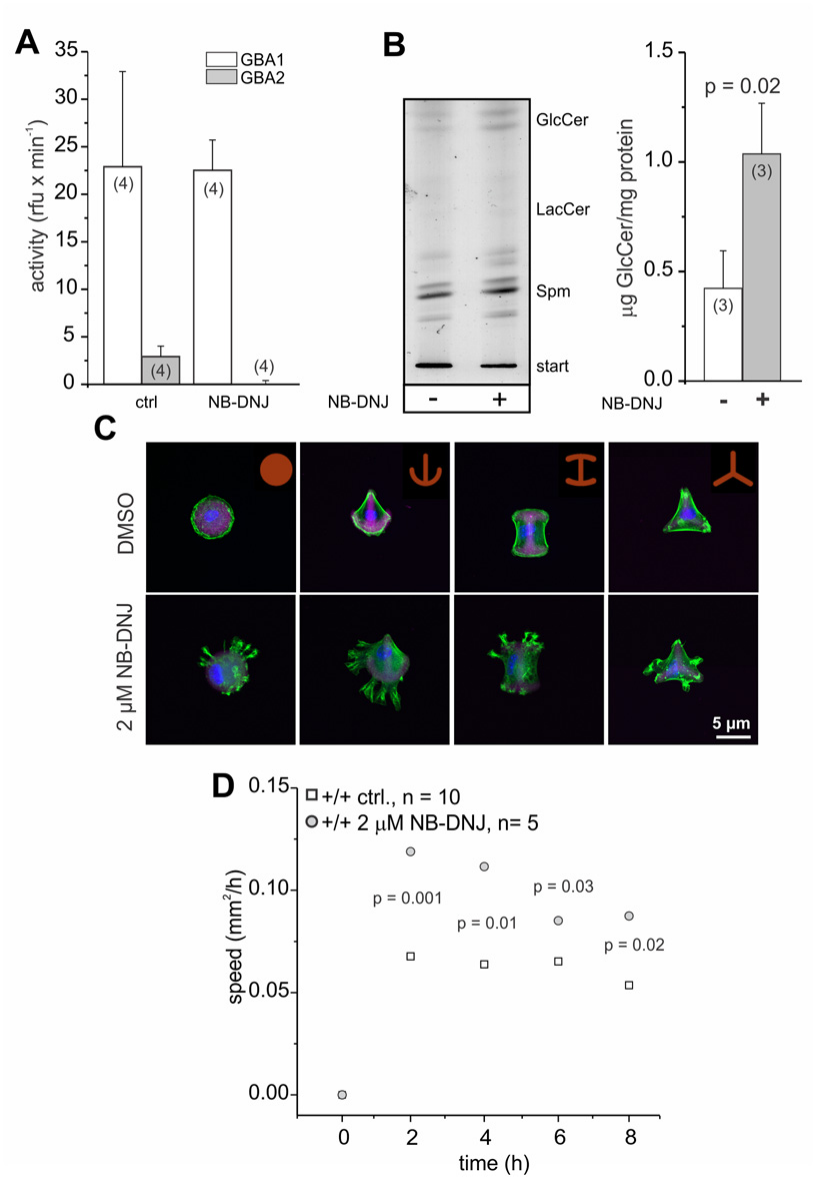
NB-DNJ treated fibroblasts also display cytoskeletal defects. Dermal fibroblasts have been treated for 48 h with 2 μM NB-DNJ. **(A)** GBA activity at pH 4 (GBA1) and pH 6 (GBA2). Beta-glucosidase activity was measured using 1.67 mM of the artificial substrate 4-methylumbelliferyl-beta-D-glucopyranoside. Data are presented as mean ± S.D. N numbers are indicated. **(B)** Lipid accumulation. Left: Thin layer chromatography (TLC) analyzing glycosphingolipids from wild-type fibroblasts in the presence (+) and absence of (-) of 2 μM NB-DNJ. Representative TLC analysis for neutral sphingolipids. GlcCer: glucosylceramide, LacCer: lactosylceramide, Spm: sphingomyelin. Right: Quantification of GlcCer. Data are presented as mean ± S.D.; n numbers and p values using One-Way ANOVA are indicated. **(CF**luorescent labeling of F-actin in dermal fibroblasts. Cells were seeded on CYTOO chips on micropatterns that are coated with fluorescently-labeled fibronectin (purple). F-actin was stained using Alexa Fluor Phalloidin 488 (green) and the DNA was stained with DAPI (blue). Scale bars are indicated. **(D)** Wound-healing assay to analyze cell migration. Average data points for wild-type dermal fibroblasts at different time points are shown; n numbers and p values using One-Way ANOVA are indicated.

To analyze whether the cellular defects observed in GBA2 knockout-fibroblasts are solely due to the loss of GBA2 and, thereby, accumulation of GlcCer, we independently assessed the contribution of GBA2 in controlling cytoskeletal dynamics by incubating wild-type fibroblasts with the GBA2 inhibitor NB-DNJ [45]. Treatment of fibroblasts for 48 h with 2 μM NB-DNJ abolished GBA2 activity (Fig. 8A). In turn, GlcCer accumulated (Fig. 8B). Similar to GBA2 knockout-fibroblasts, wild-type fibroblasts treated with NB-DNJ showed a striking difference in the organization of the F-actin cytoskeleton (Fig. 8C). We also analyzed the migration of cells treated with NB-DNJ. Similar to GBA2 knockout-fibroblasts, fibroblasts treated with NB-DNJ migrated faster than non-treated cells (Fig. 8D). Thus, inhibition of GBA2 has a similar effect on cytoskeletal dynamics and cellular behavior as genetically ablating GBA2.

### Loss of GBA2/accumulation of GlcCer alters lipid packaging and membrane fluidity

**Fig 9:**
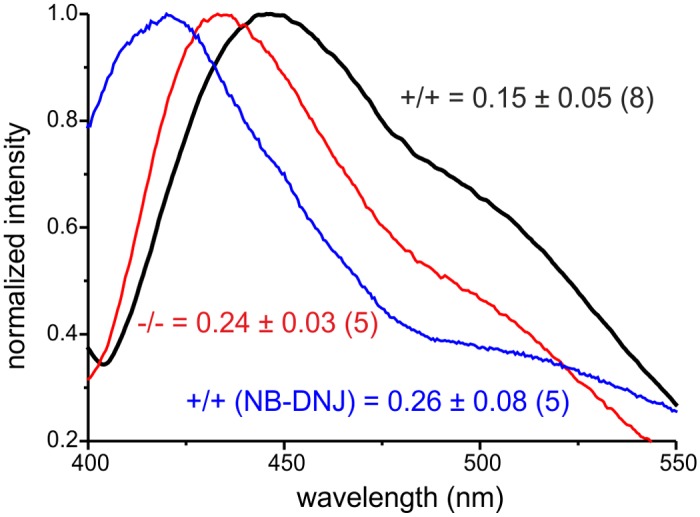
Accumulation of GlcCer alters lipid packaging of the plasma membrane Analysis of lipid packaging in GPMVs isolated from wild-type (+/+, black line), GBA2 knockout-fibroblasts (-/-, red line), and wild-type fibroblasts treated for 48 h with 2 μM NB-DNJ (blue line). GPMVs were labeled with 5 μM laurdan and analyzed by fluorescence spectroscopy. Representative emission spectra normalized to the maximum are shown. GP index has been calculated and presented as mean ± S.D. (p = 0.001); n numbers are indicated in brackets.

The cell membrane is a lipid bilayer, in which proteins are incorporated. The lipid composition of the membrane determines its characteristics and determines the function of proteins at the membrane. GlcCer is incorporated into the inner leaflet of the plasma membrane [46]. We hypothesized that accumulation of GlcCer increases the amount of GlcCer in the plasma membrane and that this controls the function of proteins at the membrane. Thus, we analyzed lipid stacking in intact plasma membranes isolated from wild-type and GBA2 knockout-fibroblasts. Giant plasma-membrane vesicles (GPMV) were isolated from fibroblasts by chemical vesiculation [47] and analyzed using laurdan fluorescent labeling. Laurdan is a membrane dye that reports the extent of water penetration into the bilayer surface due to the dipolar relaxation effect [48]. Water penetration can be directly correlated with lipid packaging and membrane fluidity [49]. Thus, laurdan fluorescence reports lipid packaging in the membrane. For highly ordered membranes, laurdan emission peaks at around 440 nm, whereas it is shifted to longer wavelengths in relatively disordered membranes [47]. GPMVs from wild-type and GBA2 knockout-fibroblasts were loaded with laurdan and the emission spectrum was measured (Fig. 9). The emission peak for wild-type fibroblasts was centered at around 460 nm, whereas the emission peak for GBA2 knockout-fibroblasts was shifted to shorter wavelengths at around 440 nm. This demonstrates that lipid packaging in the plasma membrane form GBA2 knockout-fibroblasts is more ordered than in wild-type fibroblasts. To quantify the shift, we calculated the generalized polarization (GP) index. The GP index in GBA2 knockout-fibroblasts was significantly higher than in wild-type fibroblasts (0.15 ± 0.06 vs. 0.24 ± 0.03, respectively; Fig. 9). We performed similar experiments with wild-type fibroblasts treated with 2 μM NB-DNJ for 48 h. The emission peak for GPMVs isolated from treated cells was also shifted to shorter wavelength with a GP index of 0.26 ± 0.07 (Fig. 9).

In summary, our data indicate that accumulation of GlcCer in the absence of GBA2 leads to a more ordered lipid packaging in the plasma membrane. This in turn seems to control the activity of proteins incorporated in or associated with the plasma membrane, resulting in an increase in actin and microtubule polymerization. *In vivo*, this defect is most prominent in the testis, resulting in malformation of the acrosome and the sperm head and, thereby, in male infertility.

## Discussion

Spermatogenesis is a complex process that relies on the interaction between germ and Sertoli cells. Our study reveals that the glycosphingolipid GlcCer regulates cytoskeletal dynamics in germ and Sertoli cells. Accumulation of GlcCer due to the lack of GBA2 activity induces actin polymerization and microtubule persistence, resulting in globozoospermia and male infertility.

The role of GlcCer in controlling cytoskeletal dynamics has not been established so far. However, it has been shown that ceramide, in particular C16 ceramide, promotes actin polymerization in mouse embryonic stem cells (ESCs) through PKC (protein kinase C) activation and FAK (focal adhesion kinase)/paxillin-dependent N-WASP/Cdc42/Arp2/3 complex formation [50]. This, in turn, increases cell migration. Ceramide serves as a building block for glycosphingolipids with GlcCer being one of them. Thus, the cellular changes observed after increasing C16 ceramide levels could also be due to an increase in GlcCer levels. Indeed, in GBA2 knockout-fibroblasts, we observed an increase in actin polymerization—similar to what has been described for ESCs treated with C16 ceramide [50]. Regulation of actin polymerization by lipids has also been shown for phospholipids using PIP_2_-containing vesicles [51]. In particular, PIP_2_ controls actin dynamics in highly polarized cells like T cells and neurons [52–54]. Here, PIP_2_ accumulates in membrane microdomains, where it directly regulates protein function and recruits proteins to form signaling complexes at the plasma membrane.

Not only phospholipids, but also glycosphingolipids accumulate in microdomains, where they act as signaling domains involved in recognition processes [55]. GlcCer is a structural element in the inner leaflet of the plasma membrane, but can also be incorporated into the outer leaflet of the plasma membrane. It has been suggested that GlcCer at the cytosolic surface of the plasma membrane forms signaling domains by recruiting specific proteins, thereby, controlling e.g. vesicle budding [46]. Using the environment-sensitive dye laurdan in GPMVs, we demonstrated that accumulation of GlcCer results in a more ordered lipid packaging of the plasma membrane. Future studies will reveal whether this occurs in a localized or uniform manner.

A change in lipid composition and/or lipid packaging of the membrane might also directly control protein function. For the EGF receptor (epidermal growth factor receptor), it has been demonstrated that the ganglioside GM3, surrounding the receptor in the membrane, regulates the allosteric transition from an inactive to an active EGF receptor dimer even in the absence of the ligand [56]. Furthermore, it has been shown that GlcCer controls the activity of a V-type ATPase in melanocytes [57]. Our results suggest that GlcCer accumulation in the plasma membrane controls the function of proteins like Cdc42, thereby, regulating cytoskeletal dynamics.

The role of GlcCer during spermatogenesis has been enigmatic so far. Our results suggest that GBA2 is predominantly expressed in Sertoli cells. However, more experiments are needed to clarify the physiological function of GBA2 during sperm development. In particular, Sertoli-cell specific knockout-mice will allow to unravel the role of GBA2 in Sertoli cells. In addition, analyzing the expression of GBA2 in different cell types during germ cell development will help to further understand GBA2 function in the testis.

Our results demonstrate that the GBA2 substrate GlcCer accumulates in both, Sertoli cells and sperm in GBA2 knockout-mice. Similar to our observations in dermal fibroblasts, cytoskeletal structures were also altered in Sertoli cells and sperm. Sertoli cells are polarized cells that are connected to germ cells via the ES at the apical end [20,27]. This allows to exchange proteins and lipids between these two cell types [58]. Thus, GlcCer could accumulate in germ cells even though GBA2 is not expressed there (at least not in detectable amounts). Furthermore, one function of Sertoli cells is to ensure a sufficient availability of lipids required for proliferation of millions of germ cells per day [58]. One way for the Sertoli cells to provide lipids is by recycling the lipid content of the residual body from spermatids, which is phagocytosed during spermatogenesis [58]. GBA2 could play a major role in this recycling process and accumulation of GlcCer in Sertoli cells would be further augmented. Accumulation of GlcCer in the testis in GBA2 knockout-mice occurs after P7. This is in line with our finding that GBA2 expression and activity in wild-type testis increases after P7 during sperm development. We hypothesize that GlcCer accumulates in particular at the junction between germ and Sertoli cells, similar to what has been shown for PIP_2_ in other polarized cells like T cells or neurons, where PIP_2_ forms microdomains at the immunological synapse or at the growth cone of the axon [52–54]. In GBA2 knockout-testis, F-actin hoops in the ES and the microtubule manchette in spermatids were majorly disrupted. F-actin hoops were misaligned around the developing sperm head and the microtubule manchette was more elongated. Both structures have been shown to be crucial for shaping the sperm head and forming the acrosome [35]. Interestingly, in GBA2 knockout-fibroblasts, actin polymerization was augmented and microtubule persistence was increased. These results indicate that lack of GBA2 and the concomitant accumulation of GlcCer affect similar signaling pathways in different cell types, suggesting that the regulation of cytoskeletal dynamics by GlcCer is a general mechanism.

Glycosphingolipids not only play a role in the plasma membrane, but also in membranes of organelles. In the Golgi and endosomes, glycosphingolipids have been shown to form microdomains that segregate membrane proteins and drive their sorting [59]. Furthermore, it has been proposed that GlcCer controls vesicle budding from the TGN [46]. The acrosome is formed by fusion of proacrosomal vesicles from the TGN. Thus, accumulation of GlcCer in sperm might disturb acrosome formation by inhibiting vesicle budding from the TGN and vesicle fusion. Indeed, vesicle fusion in GBA2 knockout-mice was disturbed during the cap and acrosome phase of acrosome formation, suggesting that GlcCer controls acrosome formation by regulating vesicle fusion.

In summary, our study reveals that GlcCer is a key regulator of cytoskeletal dynamics. This is particularly important during sperm development, opening up new avenues in understanding the molecular mechanisms underlying male infertility.

## Material and Methods

### Ethics statement

All animal experiments were in accordance with the relevant national and international guidelines and regulations. Animal procedures were approved by the local authorities (LANUV NRW). The generation of GBA2 knockout-mice has been described elsewhere [1].

### Isolation of sperm and male germ cells

Sperm were isolated by incision of the cauda epididymis in modified TYH medium containing 138 mM NaCl, 4.8 mM KCl, 2 mM CaCl_2_, 1.2 mM KH_2_PO_4_, 1 mM MgSO_4_, 5.6 mM glucose, 0.5 mM sodium pyruvate, 10 mM L-lactate, pH 7.4. After 15 min swim out at 37°C and 5% CO_2,_ sperm were counted. All subsequent experiments were performed at room temperature, unless otherwise stated.

For isolation of germ cells, testes were decapsulated and incubated in 1 ml Hank’s Balanced Salt Solution (HBSS) (20 mM HEPES, 137 mM NaCl, 5.4 mM KCl, 0.3 mM Na_2_HPO_4_, 0.4 mM KH_2_PO_4_, 1.2 mM MgSO_4_, 1.3 mM CaCl_2_, 6.6 mM sodium pyruvate, 0.05% lactate, 5.6 mM glucose, pH 7.2) containing 0.5 mg/ml collagenase type IA (Sigma) for 30 min at 32°C. The dissociated interstitial cells were removed by two washing steps with HBSS. The seminiferous tubules were then incubated in 1 ml HBSS containing 0.5 mg/ml Trypsin type XIII (Sigma) and 1 μg/ml DNase I (Applichem) for 10 min at 32°C. Cell aggregates were sheared gently with a Pasteur pipette. The dispersed seminiferous cells were washed twice by centrifugation at 200 x g for 5 min at room temperature. The final cell pellet was re-suspended in HBSS and filtered through a Nylon mesh (40 μm mesh).

### Isolation of P7 Sertoli cells

Seminiferous tubules were isolated from testes of 7 days old mice (P7) by removal of the *tunica albuginea*. The tubules were treated with 1 mg/ml collagenase (Sigma) at 37°C in a shaker for 8 min. The digestion was stopped by addition of DMEM/GlutaMax medium (Invitrogen) containing 10% FCS (Biochrom). The cell suspension was centrifuged at 400 x g for 8 min, the pellet was re-suspended in medium containing 0.5 mg/ml trypsin and 0.22 mg/ml EDTA (Sigma), and incubated in a shaker at 37°C for 5 min. The reaction was stopped by adding medium. The cell suspension was then treated with 1 μg/ml DNase I (Applichem) in a shaker at 37°C for 5 min. The cells were centrifuged at 600 x g for 10 min, and re-suspended in medium containing 70 U/ml penicillin, 70 μg/ml streptomycin, 100 mM sodium pyruvate, and 200 mM L-glutamine (all Life technologies). Cells were seeded at a density of 5 x 10^4^ cells/5 cm cell culture plate (Greiner bio-one) and used on the 5^th^ day for experiments. The purity of the cell population isolated using this protocol has been analyzed using immunocytochemistry using markers for Sertoli cells (beta-tubulin III, Sox9)—the preparation contains ca. 80% Sertoli cells.

### Isolation of dermal fibroblasts

Dermal fibroblasts were isolated from mouse tails using collagenase digestion. Tail pieces were incubated in DMEM/GlutaMax containing 10% FCS, 100 mM sodium pyruvate, 200 mM L-glutamine, 70 I.U./ml penicillin, 70 μg/ml streptomycin, 0.1 mg/ml collagenase (Sigma) for 3 h, 37°C, and 5% CO_2_. After digestion, the supernatant was centrifuged for 5 min, 600 x g at room temperature. The cell pellet was re-suspended in DMEM medium, cells were plated on cell culture plates, and cultured at 37°C, 5% CO_2_. After 24 h, the medium was changed.

### Antibodies and dyes

Primary antibodies: 4A12 (rat, ICC: 1:20), 2F8 (rat, WB: 1:50), pcGBA2 (rb, ICC/IHC: 1:2,000) [1,45], calnexin (Sigma #C4731, WB: 1:20,000), beta-tubulin-CY3 (Sigma #C4585, ICC: 1:200), beta-tubulin (Sigma T4026, WB: 1:1,000), HA (Roche #11867431001, WB: 1:10,000), beta-tubulin III (HISS Diagnostics, MMS-435P, ICC: 1:500, WB: 1:1,000).

Secondary antibodies: WB: IRDye680 and IRDye800 antibodies (LI-COR, 1:20,000); ICC: fluorescently-labeled antibodies (Dianova, 1:500).

Dyes: Alexa Fluor 488 Phalloidin (Molecular Probes, #A12379, ICC: 1:500), MitoTracker (Molecular Probes, M22426, ICC: 0.5 μM), peanut lectin (Sigma, #7381, ICC: 1:100), DAPI (Molecular Probes, D1306, ICC: 1:10,000).

### Immunohistochemistry (IHC) and—cytochemistry (ICC)

Testes were fixed overnight with 4% paraformaldehyde/PBS, cryo-protected in 10 and 30% sucrose, and afterwards embedded in TissueTec (Sakura Finetek). Sperm were immobilized on microscope slides and fixed for 10 min. To block unspecific binding sites, frozen sections and sperm were incubated for 1 h with blocking buffer (0.5% Triton-X 100 and 5% ChemiBLOCKER (Millipore) in 0.1 M phosphate buffer, pH 7.4). Fibroblasts, Sertoli, and germ cells were fixed for 10 min. Primary antibodies were diluted in blocking buffer and incubated overnight. Fluorescent secondary antibodies were diluted in blocking buffer containing 0.5 μg/μl DAPI (Invitrogen) and pictures were taken on a confocal microscope (Olympus FV1000). For the analysis of cytoskeletal structures in dermal fibroblasts, cells were seeded on multi-pattern fibronectin-coated CYTOO chips (#10–900–13–06, CYTOO Cell Architects).

### Protein preparation

All steps were performed at 4°C in the presence of mammalian protease inhibitor cocktail (Sigma Aldrich). Tissues or cells were homogenized in a 10-fold surplus (v/w) of hypotonic buffer (10 mM HEPES, 0.5 mM EDTA, pH 7.4) by using an Ultra-turrax (IKA) and three pulses (20 s each) of sonification (Branson sonifier). The suspension (total lysate) was centrifuged for 20 min at 1,000 × g. The supernatant (PNS, post-nuclear supernatant) was used for activity assays or Western-blot analysis.

### Fluorescence-based GBA activity assays

The assay has been performed as described previously [45]. Briefly, cleavage of 4-methylumbelliferyl(MU)-beta-D-glucopyranoside (Sigma Aldrich) was monitored in real-time in a Fluostar Omega reader (BMG labtech) at 29°C using the filter pair 355 nm/460 nm for excitation and emission, respectively. The assays were performed in 384-well plates (Greiner) in the plate mode. Per well, 25 μl of lysate containing 20 μg of total protein were used. To discriminate between GBA1 and GBA2 activity, 30 μM CBE (Conduritol B epoxide, Sigma Aldrich), an inhibitor for GBA1, or 10 μM NB-DNJ (N-butyldeoxynojirimycin, Sigma Aldrich), an inhibitor for GBA2, were included. The pH of the protein lysates and the 4-MU-beta-D-glucopyranoside solution were adjusted by diluting with McIlvaine buffer. The assay was initiated by adding 5 μl of 4-MU-beta-D-glucopyranoside (10 mM) resulting in a final concentration of 1.67 mM. The hydrolysis of 4-MU-beta-D-glucopyranoside was monitored and recorded as a change of relative fluorescence units (rfu) per minute. Each analysis was performed as a quadruplicate in parallel. Per genotype, tissues or cells from three animals were analyzed if not otherwise stated.

### Western-blot analysis

All samples were heated for 5 min at 95°C prior to separation on SDS-PAGE. For Western-blot analysis, proteins were transferred onto PVDF membranes and probed with antibodies by using the Odyssey Imaging System (LI-COR).

### Transfection

1x10^6^ mouse fibroblasts were re-suspended in 100 μl of transfection buffer (Neon transfection system, Life technologies) and 4 μg of plasmid DNA was added. Using a microporator mini (Digital Bio Technology, MP-100), 10 μl of the cell suspension were subjected to two pulses (20 ms each) of 1000 V and afterwards transferred to Poly-L-Lysine-coated glass-bottom dishes (Mat Tek, #P35G-1.5–20-C). A total of 30 μl of cells were electroporated. The cells were allowed to grow overnight at 37°C and 5% CO_2_ in medium.

### Live-cell imaging

Cells were imaged 24 h after transfection using the DeltaVision Core microscope (Applied Precision, Inc.). Images were acquired every 3 s for 200–500 ms over 5 min and image analysis was done using Metamorph (version 7.0, Molecular Devices Corporation) using the track-points function. Microtubule tracks were followed from the first frame an EB3-labeled microtubule plus-tip appeared until the last frame (when the plus-tip was no longer visible). Data for velocity (microtubule growth-rate) and distance (microtubule persistence) were calculated. At least 10 microtubule tracks were followed in each cell, and at least 7 cells per cell line and genotype were analyzed.

### Identification of mGBA2 by mass spectrometry

For GeLCMS, proteins of Sertoli cells, testis, and sperm were separated on SDS gels and stained with Coomassie. Per lane, 14–17 gel slices were excised, proteins were in-gel digested with trypsin (Promega), peptides were separated in a 90 or 180 min gradient by a nanoAcquity LC System equipped with a HSS T3 analytical column (1.8 μm particle, 75 μm x 150 mm) (Waters), and analyzed by ESI-LC-MS/MS using an LTQ Orbitrap Elite mass spectrometer (Thermo Scientific). All database searches were performed using SEQUEST as well as MS Amanda (Mechtler lab, Vienna, Austria) algorithm, embedded in Proteome Discoverer (Rev. 1.4, Thermo Electron 2008–2011), with a NCBI protein database (mouse, accession number NP_766280.2, accessed June 13, 2013). Only fully tryptic peptides with up to two missed cleavages were accepted. Oxidation of methionine was permitted as variable modification. The mass tolerance for precursor ions was set to 10 ppm; the mass tolerance for fragment ions was set to 0.4 amu. To filter the results, a peptide FDR threshold of 0.01 (q-value) according to Percolator was set in Proteome Discoverer; two peptides per protein and peptides with search result rank 1 were required.

### Lipid analysis using thin-layer chromatography (TLC)

For lipid extraction, dermal fibroblasts from GBA2 wild-type (+/+) and knockout-mice (-/-) were grown until confluency, washed once in PBS, and harvested using trypsin/EDTA in medium. Cells were pelleted for 7 min at 700 x g and room temperature. Afterwards, cells were lysed in 1 ml bi-destilled water with three pulses (30 s each) of sonification (Branson sonifier). Lipids were extracted for 24 h at 37°C in chloroform/methanol/water (10/5/1, v/v/v). For a better analysis of glucosylceramide, glycerophospholipids were degraded by alkaline hydrolysis with 125 mM sodium hydroxide for 2 h at 37°C. After neutralization with acetic acid, lipid extracts were desalted by reversed-phase chromatography and separated into acidic and neutral glycosphingolipids as described previously [60,61].

For separation of neutral lipids by thin layer chromatography (TLC), lipids derived from an extract of 1–1.5 mg of total protein were applied to prewashed thin layer Silica Gel 60 (Merck, Darmstadt, Germany) and chromatograms were developed and quantified as described previously [61].

### Extraction and quantification of sphingolipids using mass spectrometry

Sperm cells, testis, and Sertoli cells were frozen in liquid nitrogen and ground to a fine powder using the Precellys24 tissue homogenizer (PeqLab). Lipids were extracted and fractionated using solid-phase-extraction on silica columns [62]. Long chain bases (LCB), ceramides (Cer), and hexosylceramides (HexCer) were eluted with acetone/2-propanol (9/1, v/v) and sphingomyelin (Spm) was eluted with methanol. The purified sphingolipids were analyzed via direct infusion nanospray mass-spectrometry using an Agilent 6530 Accurate-Mass Q-TOF LC/MS device [62]. Sphingolipids were quantified after collision-induced dissociation by scanning for specific fragment ions: LCB, neutral loss of 18.0106; Cer and HexCer, precursor ion scanning for m/z 266.2842 (d18:0), m/z 264.2686 (d18:1) and m/z 262.2493 (d18:2); Spm, precursor ion scanning for m/z 184.0739. Internal standards were added for each sphingolipid class [63].

### G-/F-Actin assay

The assay was performed according to the manufacturer’s protocol (#BK037, Cytoskeleton). In brief, cells were lysed in a detergent-based lysis buffer, which solubilizes G-actin but also stabilizes and maintains F-actin. As a control, protein lysates were treated with an F-actin polymerizing solution for 15 min at 37°C. Equal volumes of each samples were subjected to ultracentrifugation (100,000 x g, 1 h) to separate F-actin from G-actin. F-actin was maintained in the pellet fraction, whereas G-actin was maintained in the supernatant. The pellet was dissolved in F-actin depolymerizing buffer and incubated on ice for 1 h. Samples were run on a SDS-PAGE and analyzed by Western blot. Ratios of F-actin/G-actin for each genotype were calculated as percentage of the control.

### Analysis of actin structures

Mouse fibroblasts were plated on Cytoo chips (Cytoo Cell Architects, # 10–900–13–06) placed in a 35 mm cell culture plate. Cells were fixed with 4% paraformaldehyde and labeled with Alexa Fluor 488 Phalloidin and DAPI. Images were taken using an Olympus FV1000 confocal microscope. Filopodia (slender actin-protrusions) and lamellipodia (wave-like actin extensions) structures were counted.

### Wound-healing assay

Silicone cell culture-inserts (Ibidi) with a defined cell-free gap (width = 500 μm) were placed in 35 mm cell culture dishes. 4 x 10^4^ cells were transferred into each of the culture inserts and incubated at 37°C, 5% CO_2_ for 2 h. Afterwards, inserts were removed and cells were washed with PBS. Fresh medium was added and a phase contrast image was taken (t = 0 h) using the Nikon eclipse (TE 2000-S) microscope. An image of the same region was taken every 2 hours (t = 2, 4, 6, 8 h). The area of the cell-free gap was measured using ImageJ (version 1.46m) and the speed of migration was calculated.

### Isolation of GPMVs

Giant plasma-membrane vesicles (GPMVs) were isolated as described elsewhere [47]. In brief, dermal fibroblasts were incubated with GPMV buffer (10 mM HEPES, 150 mM NaCl, 2 mM CaCl_2_, pH 7.4) containing 2 mM NEM for 1–2 h at 37°C, 5% CO_2_. The supernatant was centrifuged for 10 min at 2000 x g and room temperature to pellet cell debris and intact cells. The resulting supernatant was subjected to high-speed centrifugation for 1 h at 20,000 x g and 4°C to pellet the vesicles. The pellet was re-suspended in GPMV buffer.

### Fluorescence spectroscopy

Measurements were performed in a quartz cuvette using the FluoroMax-4 Spectrofluorometer. The emission spectrum was recorded from 400 to 500 nm at 385 nm excitation to detect the lipid resonance-peak at 425 nm. All samples were normalized to the lipid resonance-peak for the GPMV buffer. GPMVs were labeled with 5 μM laurdan (6-Dodecanoyl-2-Dimethylaminonaphthalene, Molecular Probes, #D250) for 20 min at 23°C. Measurements were performed at 350 nm excitation and fluorescence emission was recorded from 400 to 600 nm. All measurements were done at 23°C. The GP value was calculated according to the following equation:

$$\text{GP}=\frac{\sum_{420}^{460}{\text{I}}_{\text{x}}-\sum_{470}^{510}{\text{I}}_{\text{x}}}{\sum_{420}^{460}{\text{I}}_{\text{x}}+\sum_{470}^{510}{\text{I}}_{\text{x}}}$$

## Supporting Information

S1 Fig Protein sequence mGBA2. Peptides identified by mass spectrometry are indicated. Red: peptides found in P7 Sertoli cells; blue: peptides found in testis.
(DOCX)
