# Supplementary figures and images for: Accumulation of Glucosylceramide in the Absence of the Beta-Glucosidase GBA2 Alters Cytoskeletal Dynamics
Source: PLoS Genet. 2015 Mar 24;11(3):e1005063. doi: 10.1371/journal.pgen.1005063 (PMC4372435; doi:10.1371/journal.pgen.1005063)

**Supporting Information**


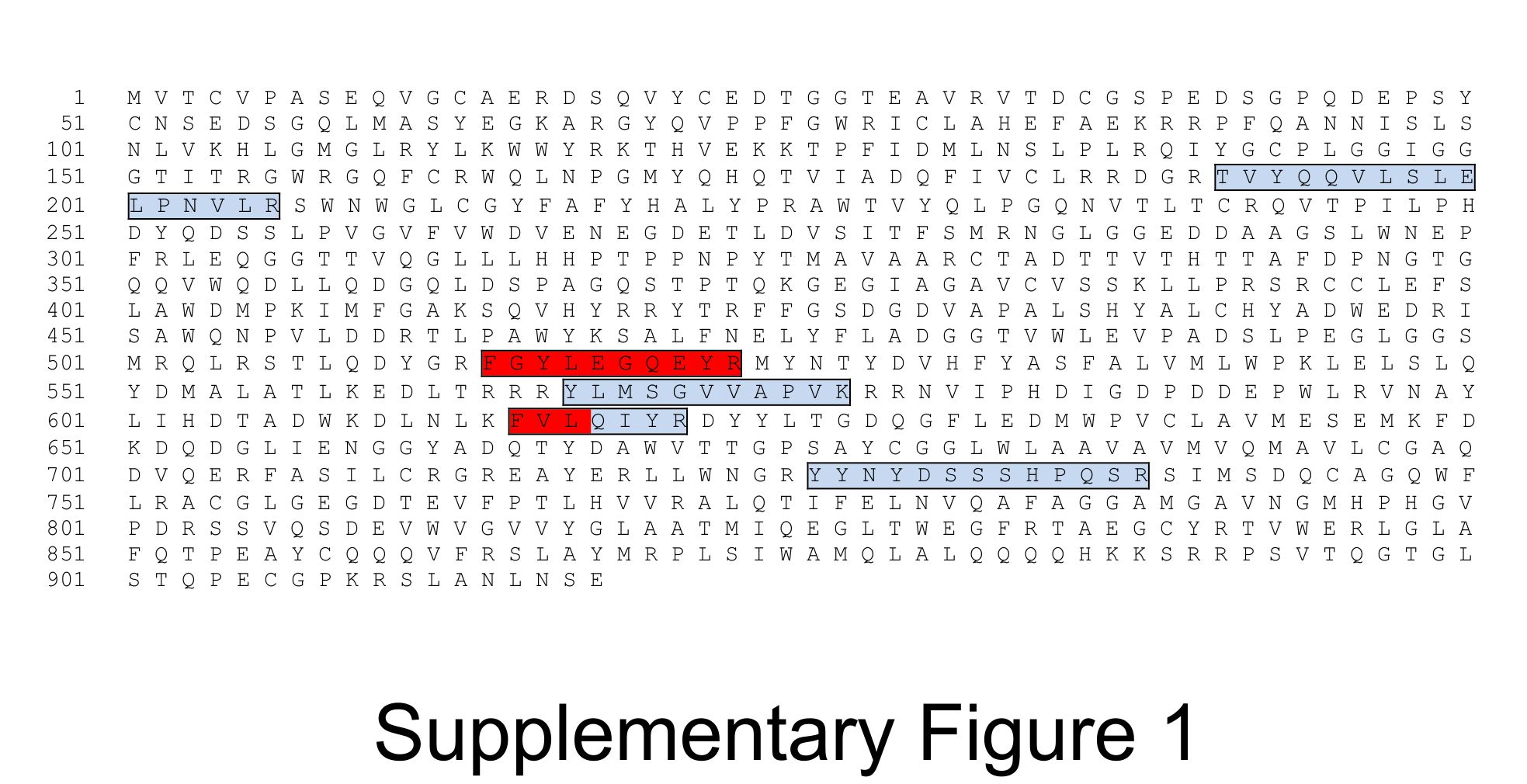


**Supporting Figure 1**

Supplement: S1 Fig — Peptides identified by mass spectrometry are indicated. Red: peptides found in P7 Sertoli cells; blue: peptides found in testis. (DOCX) [file pgen.1005063.s001.docx]
